# Fluoroquinolone susceptibility in first-line drug-susceptible *M. tuberculosis* isolates in Lima, Peru

**DOI:** 10.1186/s13104-021-05832-0

**Published:** 2021-11-14

**Authors:** Alvaro Schwalb, Rodrigo Cachay, Ericka Meza, Tatiana Cáceres, Amondrea Blackman, Fernanda Maruri, Timothy R. Sterling, Eduardo Gotuzzo

**Affiliations:** 1 Instituto de Medicina Tropical Alexander Von Humboldt, Universidad Peruana Cayetano Heredia, Av. Honorio Delgado 430, San Martín de Porres, 15102 Lima, Peru; 2 Vanderbilt Tuberculosis Center, Vanderbilt University School of Medicine, Nashville, TN USA

**Keywords:** Levofloxacin, Moxifloxacin, Ofloxacin, Drug resistance

## Abstract

**Objective:**

To determine at two distinct time points the prevalence of resistance to ofloxacin (OFX), the representative class drug of fluoroquinolones (FQs), in *M. tuberculosis* isolates susceptible to first-line drugs.

**Results:**

There were 279 *M. tuberculosis* isolates from the two cohorts (2004–2005: 238 isolates; 2017: 41 isolates) that underwent OFX drug-susceptibility testing (critical concentration: 2 µg/ml). Of 238 isolates in Cohort 1, no resistance to OFX was detected (95% CI 0–0.016); likewise, in Cohort 2, no resistance to OFX was detected in 41 isolates (95% CI 0–0.086). Our findings suggest that FQ use remains a viable option for the treatment of first-line drug-susceptible TB in Peru.

## Introduction

Tuberculosis (TB) can be cured with effective multi-drug therapy. However, drug intolerance or antibiotic resistance necessitate alternatives to current first-line antimicrobials [1]. Fluoroquinolones (FQ), a family of broad-spectrum antibiotics (ciprofloxacin (CIPRO), levofloxacin (LVX), moxifloxacin (MOX), ofloxacin (OFX)), have bactericidal activity against *M. tuberculosis* (*Mtb*) by inhibiting DNA gyrase, which blocks supercoiling and movement of replication forks [1]. Third-generation FQs such as LVX and MOX have greater activity against *Mtb* than earlier-generation FQs, with MOX exhibiting the highest activity albeit at a higher cost [1, 2]. Current WHO guidelines recommend the use of LVX or MOX in multidrug-resistant TB (MDR-TB) treatment regimens, as their use leads to a decreased risk of treatment failure or relapse, and death [3]. FQs are also used to treat drug-susceptible TB when there is hypersensitivity or toxicity (especially hepatotoxicity) to first-line drugs [1, 4]. MOX is also part of a multidrug regimen that effectively treats drug-susceptible TB in 4 months [5].

FQs are widely used for diseases other than TB. In 2002, they became the most commonly prescribed antibiotic class in adults in the United States [6]. Of note, FQs are available without prescription in several countries, including Peru [7]. A steady increase in the use of FQs for community-acquired respiratory infections may partially improve symptoms due to TB and delay the start of appropriate TB treatment [8]. FQ monotherapy can ultimately lead to FQ cross-resistance; DNA gyrase is the only target for FQs in *Mtb*, thus a single mutation is sufficient to cause resistance [1]. The rate of FQ-resistance among first-line drug-susceptible has not been clearly established in Peru because FQ-resistance testing is not routinely performed. The aim of this study was to determine the prevalence of resistance to OFX (the representative class drug) in isolates with first-line drug-susceptible *M. tuberculosis* at two distinct time points.

## Main text

### Materials and methods

We assessed OFX-resistance in *Mtb* isolates from sputum samples of TB patients from San Juan de Lurigancho (SJL), a TB high-burden district in Lima, Peru. Over 50% of TB and 60% of MDR-TB cases in Peru are reported in Lima, of which, SJL has among the highest TB incidence, reporting over 100 pulmonary TB cases per 100,000 inhabitants annually [9]. Second-line drug-susceptibility testing (DST) is not routinely performed for first-line drug-susceptible *Mtb* samples. Isolates from first-line drug-susceptible *Mtb* isolates conducted in SJL during the periods May–August 2004 and May–August 2005 (Cohort 1), and June-October 2017 (Cohort 2) were available for FQ DST. Patients were recruited prospectively as part of studies to evaluate novel TB diagnostic methods. Patients were enrolled from their primary care facility prior to treatment initiation, and all provided written informed consent, authorizing the use of stored samples for future TB studies. The study was approved by the Institutional Ethics Committee of Universidad Peruana Cayetano Heredia (SIDISI:200817) and Vanderbilt University Medical Center (IRB#081130).

All isolates had previously undergone DST for first-line TB drugs performed by agar proportion method and underwent DST for FQs for this study. Isolates were cultured using BD-BBL^TM^MGIT™ supplemented with BBL^TM^MGIT^TM^OADC enrichment and confirmed as *Mtb* complex using Capilia TB-Neo. For Cohort 1, 200 µl of the liquid culture suspension was used to inoculate Löwenstein-Jensen slants. When positive, DST by agar proportion method using 7H10 Media was performed. A suspension adjusted to a McFarland standard of 1 was prepared and further diluted to 1:5 with distilled sterile water. Then 100 µl was inoculated into each quadrant. OFX and MOX were dissolved in 0.1 N NaOH solution and in sterile distilled water, respectively. Drugs were added to the agar plate for final concentrations: 2.0 µg/ml for OFX and 0.5 µg/ml for MOX. For internal quality control, we used *Mtb* H37Rv with each new batch of media prepared. Additionally, a random subset of 60 isolates was selected and sent to the TB Research Laboratory at Vanderbilt University Medical Center and underwent repeat testing by BACTEC MGIT-960 and agar proportion method using the aforementioned drug concentrations. For cohort 2, DST by BACTEC MGIT-960 SL-DST was performed using a drug concentration of 2.0 µg/ml for OFX and 1.5 µg/ml for LVX.

### Results

Of 279 patients, 228 (82%) had not previously been treated for TB. The median age was 29 years (IQR: 23–39), and 153 (55%) were male. There were no significant differences in median age or male/female ratio between cohorts (p = 0.69 and p = 0.86, respectively). All *Mtb* isolates were susceptible to first-line drugs and there was no FQ-resistance detected in either cohort. Of 238 isolates in Cohort 1, no resistance to OFX or MOX was detected (95% CI 0–0.016). Likewise, in Cohort 2, there was no resistance to OFX or LVX detected in 41 isolates (95% CI 0–0.086) (Table 1). The 60 isolates tested at Vanderbilt University Medical Center were also OFX-susceptible (95% CI 0–0.06).

**Table 1:** Fluoroquinolone DST among first-line drug-susceptible *M. tuberculosis* isolates

	Cohort 1^a^ (n = 238)		Cohort 2^b^ (n = 41)	
	OFX	MOX	OFX	LVX
Susceptible	238	238	41	41
Resistant	0	0	0	0

OFX: Ofloxacin; MOX: Moxifloxacin; LVX: Levofloxacin

^a^Cohort 1: May to August 2004 and May to August 2005

^b^Cohort 2: June to October 2017

### Discussion

Our study found no OFX-resistance in two cohorts of first-line drug-susceptible *Mtb* isolates collected 13 years apart. The demographic data of the participants was similar to that of TB patients throughout Peru; almost half of all cases are male and young (15 to 34 years old) [9]. Previous studies have attempted to measure the variation of FQ-resistance trends over time; one study from Korea reported no changes in the resistance profile over 5 years, with an FQ-resistance average of 0.8% among non-MDR-TB isolates, despite reported use of FQs before TB diagnosis [10]. Nonetheless, studies have concluded that prior exposure to FQs, especially in a setting where they can be given without prescription, is associated with FQ-resistance [8, 11]. More than 10 days of FQ-exposure has been associated with a seven-fold higher risk of resistance compared with non-exposure [12]. Although, FQ resistance rates in drug-susceptible TB are usually low [10, 12], caution is required since monotherapy can lead to resistance to not only the FQ prescribed, but also the entire class of FQ, limiting TB treatment options [13]. Also, further surveillance should be implemented to evaluate additional Mtb isolates and monitor for changes in FQ resistance rates.

FQ use prior to TB diagnosis can be as high as 23–48% among newly-diagnosed patients [14, 15]. FQ-resistance is associated with worse treatment outcomes of MDR-TB than resistance to a second-line injectable drug [16]. Furthermore, FQ-exposure before TB diagnosis is independently associated with death, after adjusting for age and HIV-serostatus [17]. While HIV infection is prevalent in less than 1% of the general population in Peru, TB/HIV coinfection has been estimated to be 4.9% although higher proportions are found outside Lima [9].

Apart from being the best alternative for TB drug intolerance or resistance, FQs have promise as potential first-line treatment of drug-susceptible TB. However, FQs do not have sterilizing activity; they need to be given in combination with sterilizing drugs to potentially reduce treatment duration [2]. Results from an international trial evaluating multi-drug treatment-shortening (4-month) regimens including moxifloxacin and rifapentine was non-inferior to standard 6-month treatment [5]. Treatment shortening would decrease the burden on the healthcare system and likely reduce the proportion of patients who do not complete treatment [18].

FQs remain a viable option in Peru for treating TB that is susceptible to first-line drugs. However, to ensure that this class of drugs remains a viable option for treatment of tuberculosis, antibiotic use should be regulated to limit improper, prolonged, or repeated use of FQs, and prevent the development of drug resistance.

## Limitations

One limitation of our study is that we did not have data on FQ-exposure prior to TB diagnosis. The uncertainty regarding FQ exposure is an important factor to consider as it conveys the risk of resistance and of negative outcomes. Resistance trends for early generation FQs (CIPRO) have been widely studied in Peru among gastrointestinal and urinary pathogens: *C. jejuni* (48.1%-87.4%) and *E. coli* (70.4%) [19, 20]. Unfortunately, data are scarce on LVX or MOX use, especially for respiratory tract infections. Another limitation is the relatively small sample size, especially cohort 2. Nonetheless, the absence of FQ-resistance at two separate time points is an encouraging result given that all isolates were gathered in a high-burden area with poor regulation of antibiotic prescriptions.

## Data Availability

The datasets used and/or analyzed during the current study available from the corresponding author on reasonable request.

## Abbreviations

CI: Confidence intervals
CIPRO: Ciprofloxacin
DST: Drug-susceptibility testing
FQ: Fluoroquinolones
LEV: Levofloxacin
MDR-TB: Multidrug-resistant tuberculosis
MOX: Moxifloxacin
Mtb: *Mycobacterium tuberculosis*
OFX: Ofloxacin
SJL: San Juan de Lurigancho
TB: Tuberculosis
